# The Transcription Regulator *YgeK* Affects Biofilm Formation and Environmental Stress Resistance in Avian Pathogenic *Escherichia coli*

**DOI:** 10.3390/ani12091160

**Published:** 2022-04-30

**Authors:** Mei Xue, Dandan Fu, Jiangang Hu, Ying Shao, Jian Tu, Xiangjun Song, Kezong Qi

**Affiliations:** 1 Jinling Institute of Technology, College of Animal Science and Food Engineering, Nanjing 211169, China; withxm@163.com; 2 Anhui Province Key Laboratory of Veterinary Pathobiology and Disease Control, Anhui Agricultural University, Hefei 230036, China; fudandan2020@126.com (D.F.); vethjg@163.com (J.H.); julieshao1005@163.com (Y.S.); tujian1980@126.com (J.T.)

**Keywords:** avian pathogenic *Escherichia coli*, transcription regulators, *ygeK*, biofilm, environmental stress

## Abstract

**Simple Summary:**

Avian pathogenic *Escherichia coli* (APEC) is the pathogen responsible for colibacillosis in poultry. Transcriptional regulator *ygeK* has been shown to decrease APEC’s flagellar formation ability, bacterial motility ability, serum sensitivity, and adhesion ability. However, we did not study the effects of *ygeK* on biofilm formation and environmental stress resistance in APEC. In this study, we investigated *ygeK* in APEC biofilm formation and bacterial resistance to different environmental stresses. We also analyzed the multi-level regulation of *ygeK* in APEC and investigated associations between differentially expressed proteins and key *ygeK* targets. This work provides a basis for further analysis of APEC pathogenesis mechanisms.

**Abstract:**

Avian pathogenic *Escherichia coli* (APEC) is one of the most common pathogens in poultry and a potential gene source of human extraintestinal pathogenic *E. coli* (ExPEC), leading to serious economic losses in the poultry industry and public health concerns. Exploring the pathogenic mechanisms underpinning APEC and the identification of new targets for disease prevention and treatment are warranted. *YgeK* is a transcriptional regulator in APEC and is localized to the type III secretion system 2 of *E. coli.* In our previous work, the transcription factor *ygeK* significantly affected APEC flagella formation, bacterial motility, serum sensitivity, adhesion, and virulence. To further explore *ygeK* functions, we evaluated its influence on APEC biofilm formation and resistance to environmental stress. Our results showed that *ygeK* inactivation decreased biofilm formation and reduced bacterial resistance to environmental stresses, including acid and oxidative stress. In addition, the multi-level regulation of *ygeK* in APEC was analyzed using proteomics, and associations between differentially expressed proteins and the key targets of *ygeK* were investigated. Overall, we identified *ygeK*’s new function in APEC. These have led us to better understand the transcriptional regulatory *ygeK* and provide new clues about the pathogenicity of APEC.

## 1. Introduction

Avian pathogenic *Escherichia coli* (APEC) is a type of extraintestinal pathogenic *E. coli* (ExPEC) that causes colibacillosis in poultry, leading to significant economic losses to the poultry industry [1]. Due to its diverse serotypes and complex virulence factors, no effective vaccines are available against APEC [2]. In addition, APEC is becoming increasingly resistant to widespread antibiotic use; even the polymyxin resistance gene *mcr-1* has begun to appear in clinical APEC isolates, with life-threatening risks on a global scale [3,4,5]. Gene cluster comparisons of animal and human *mcr*-resistant strains suggest that bacteria carrying *mcr* genes are potentially zoonotic. Therefore, exploring pathogenic mechanisms in APEC is important and will promote the development of effective vaccines or new drug targets to control this infection.

Biofilms are multicellular bacterial aggregates bound by a polymeric matrix consisting of complex mixtures of extracellular polysaccharides, proteins, and DNA [6,7]. These structures allow bacteria to tolerate a variety of environmental pressures [7,8]. Many bacterial species form complex and diverse biofilms. Researchers have concentrated on exploring the formation mechanism of biofilms and looking for inhibitory strategies for biofilm formation [9,10]. In APEC, several transcription regulators regulate bacterial biofilm formation, e.g., deletion of the two-component system *basSR* inhibits in vitro APEC biofilm formation and decreases bacterial virulence and colonization in vivo [11]. *PhoP* is a transcriptional regulator in the two-component *phoP/phoQ* regulatory system; it up-regulates APEC biofilm formation and is associated with changes in bacterial drug resistance and cell-membrane-related properties [12]. *McbR* increases APEC biofilm formation by up-regulating transcription of the biofilm-associated genes, *bcsA*, *fliC*, *wcaF*, and *fimA*, and also affects oxidative responses by regulating transcription of the yciGFE operon [13]. Oxidative stress is one of the stress environments that bacteria encounter when they infect the host. In APEC, some genes have been reported to be associated with environmental stress (such as acid, alkali, and oxidative stress). Understanding bacterial resistance to environmental stress will help further explore bacterial survival mechanisms.

*YgeK* was identified as a regulator of gene expression in enterohemorrhagic *E. coli* [14,15]. We previously showed that *ygeK* inactivation in AE81 reduced several bacterial functions, including flagella formation, motility, bactericidal activity, and adhesion [16]. Studies reported that flagella are more than a locomotive organelle for *E. coli,* they are also critical for biofilm formation [17]. However, we did not study the effects of *ygeK* on biofilm formation in APEC. In this study, we investigated *ygeK* in APEC biofilm formation and bacterial resistance to different environmental stresses. We also analyzed the multi-level regulation of *ygeK* in APEC and investigated associations between differentially expressed proteins and key *ygeK* targets. This work provides a basis for further analysis of APEC pathogenesis mechanisms.

## 2. Materials and Methods

### 2.1. Wild-Type, Mutant, and Complement Strains

The wild-type strain, AE81, was isolated from the lung of a dead, septicemic chicken with suspected colibacillosis in Anhui, China [18]. The mutant, AE81Δ*ygeK*, and complemented strain, AE81Δ*ygeK*-pCm*ygeK,* were constructed in our previous study [16]. Where necessary, we supplemented chloramphenicol (30 µg/mL) to lysogeny broth solid medium.

### 2.2. Crystal Violet (CV) Staining of Biofilms

AE81, AE81Δ*ygeK*, and AE81Δ*ygeK*-pCm*ygeK* cultures were diluted to OD_600_ = 0.03 and incubated at 28 °C for 72 h. Stationary phase cultures were washed three times in sterile phosphate buffer saline (PBS) and air-dried. Then, 100% methanol was added to immobilize biofilm-forming cells for 5 min and a 0.1% (*w/v*) CV solution was added to stain cells for 15 min. Excess stain was rinsed away with distilled water and cells were re-air-dried. The remaining CV in the growth tube was dissolved in 33% glacial acetic acid (Sanggong, Shanghai, China) and the solution read at 492 nm on a Micro Elisa microplate reader (Thermo Scientific, Pittsburgh, PA, USA) [19]. Experiments were performed three times.

### 2.3. Scanning Electron Microscopy (SEM)

We incubated 1 mL bacterial suspensions (OD_600_ = 1.0) on sterile glass coverslips (diameter = 10 mm) in 12-well plates at 37 °C for 24 h. The next day, the coverslip was washed three times in PBS and glutaraldehyde (2.5%) was added to the wells. After incubation at 4 °C for 10 h, wells were washed three times in PBS and treated with 1% osmic acid for 5 h. Cells were then dehydrated in 30, 50, 60, 70, 80, 90 and 100% ethyl alcohol. Cells were then observed using field SEM (Hitachi S-4800, Chiyotaku, Japan).

### 2.4. Hydrogen Peroxide (H_2_O_2_) Stress Assays

Overnight AE81, AE81Δ*ygeK*, and AE81Δ*ygeK*-pCm*ygeK* cultures were diluted to OD_600_ = 0.03 in fresh lysogeny broth (LB) broth. Then, 10 µL culture aliquots were spotted onto LB agar plates containing 0.8 mM H_2_O_2_ and allowed to air dry [13]. The plates were incubated at 37 °C over night, after which colonies were photographed the next day. Experiments were performed three times.

### 2.5. Acid Resistance Assays

AE81, AE81Δ*ygeK*, and AE81Δ*ygeK*-pCm*ygeK* cultures were grown to logarithmic phase (OD_600_ = 1.0) then centrifuged and resuspended in PBS. The pH of LB medium was adjusted with Tris-HCl (100 mmol/L, pH 10.0) to different acid pH levels (pH = 1, pH = 2, pH = 3). Bacteria were cultured in LB at different pH’s at 37 °C for 30 min and then aliquots were spotted onto LB agar plates and incubated overnight. The next day, colonies were counted. All experiments were performed in triplicate. 

### 2.6. iTRAQ-Based Quantitative Proteomic Analysis

The sequenced strains were the original, AE81, and the deletion strain, AE81Δ*ygeK*. Isobaric tags for relative and absolute quantitation (iTRAQ) proteomic services were provided by Shenzhen Huada Gene Co., Ltd. (Shenzhen, China). iTRAQ data were quantified by IQuant [20] software independently developed by BGI, which integrates the Mascot Percolator algorithm [21]. A 1% false-positive rate (FDR) filtering (PSM-level FDR ≤ 0.01) step at the peptide-spectrum match level was performed to obtain spectrum and peptide lists for identification, then proteins were assembled using peptides, and protein groups were generated. Proteins were filtered again using picked protein FDR [22] with FDR at 1% at the protein level (protein-level FDR ≤ 0.01) to control the false positive rate. The Kyoto Encyclopedia of Genes and Genomes Pathway (KEGG-PATH) of differentially expressed proteins was to compare the identified proteins with the Kyoto Encyclopedia of Genes and Genomes (KEGG) database to derive corresponding classification pathway results.

### 2.7. Statistical Analysis

SPSS (v19.0) software was used to analyze data. Between AE81 and AE81Δ*ygeK* groups, or AE81Δ*ygeK* and AE81Δ*ygeK*-pCm*ygeK* groups, paired t-tests were used for statistical comparisons. A *p*-value ≤ 0.05 was used to indicate statistical significance.

## 3. Results

### 3.1. YgeK Inactivation Decreases Biofilm Formation in AE81 Strains

**Figure 1:**
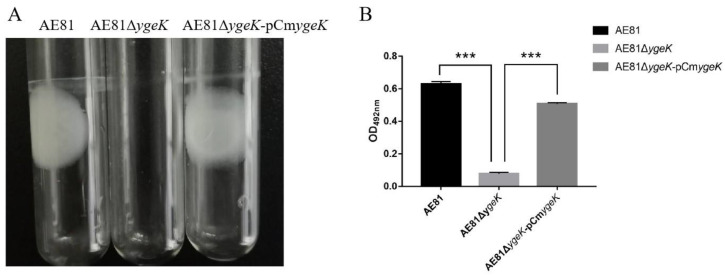
(**A**) Biofilms formed on tubes by AE81, AE81Δ*ygeK*, and AE81Δ*ygeK-*p*C*m*ygeK* strains. (**B**) Measurement of biofilm mass. Values are average of three independent experiments. Error bars indicate standard deviation (*** *p* < 0.001).

The influence of *ygeK* inactivation on biofilm formation was evaluated in vitro. AE81 and AE81Δ*ygeK*-pCm*ygeK* strains formed intact biofilms on glass tubes and liquid surfaces, but the AE81Δ*ygeK* strain generated almost none (Figure 1A). Biofilms were stained in 0.1% crystal violet, dissolved in 33% glacial acetic acid, and read at 492 nm (Figure 1B).

### 3.2. Biofilm Observations using SEM

**Figure 2:**
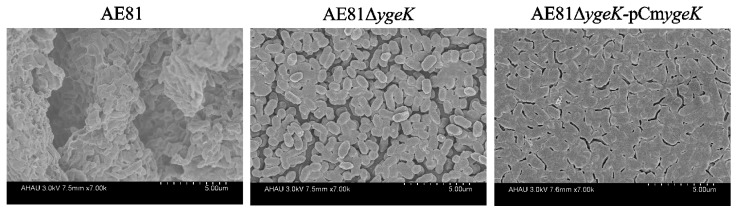
Biofilm structures were observed by scanning electron microscopy (×7000 magnification).

AE81 biofilm morphology under SEM was compact; the outer layer had a thick extracellular matrix and surface bacteria were aggregated to form a dense regional surface layer. *YgeK* deletion in AE81 resulted in thin membranous structures and large bacterial shapes in the biofilm, whereas AE81Δ*ygeK*-pCm*ygeK* biofilms were more compact than AE81Δ*ygeK* (Figure 2). Inactivation of *ygeK* resulted in reduced ability of APEC to form biofilms.

### 3.3. YgeK Influences APEC Resistance to H_2_O_2_

**Figure 3:**
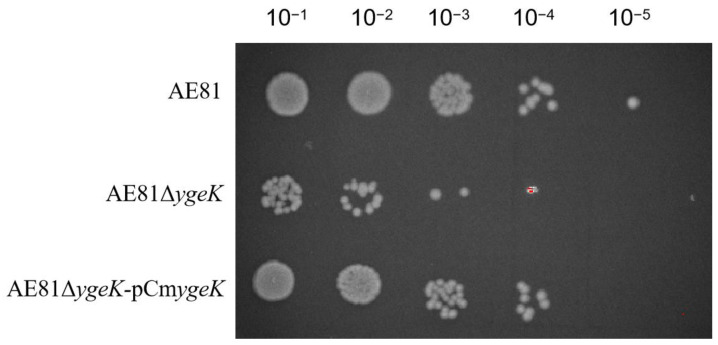
Hydrogen peroxide sensitivity in AE81, AE81Δ*ygeK*, and AE81Δ*ygeK-*p*CmygeKI* strains.

In co-cultured bacteria with H_2_O_2_ at different dilutions (10^−1^, 10^−2^, 10^−3^, 10^−4^, and 10^−5^), AE81, AE81Δ*ygeK*, and AE81Δ*ygeK-*p*CmygeK* strains were observed. However, at 10^−5^, only the AE81 strain was observed. As shown (Figure 3), AE81Δ*ygeK* colony numbers were reduced when compared with AE81 and AE81Δ*ygeK-*p*CmygeK*, suggesting inactivation of *ygeK* led to APEC’s response to H_2_O_2_ stress.

### 3.4. YgeK Influences APEC Acid Resistance

**Figure 4:**
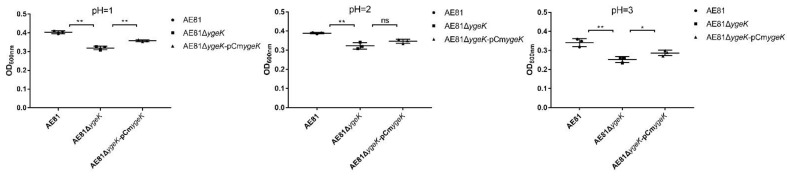
AE81, AE81Δ*ygeK,* and AE81Δ*ygeK-*p*CmygeK* resistance to different pH values (pH = 1.0, 2.0, 3.0). Values are average of three independent experiments (* *p* < 0.05, ** *p* < 0.01, ns: no significance).

Acid resistance assays are often performed to evaluate a pathogen’s resistance to acid stress environments [23,24]. As shown (Figure 4), *ygeK* inactivation reduced acid resistance significantly in medium at different pH values (pH 1.0, 2.0, and 3.0).

### 3.5. Screening Differentially Expressed Proteins in AE81 and AE81ΔygeK Strains

**Figure 5:**
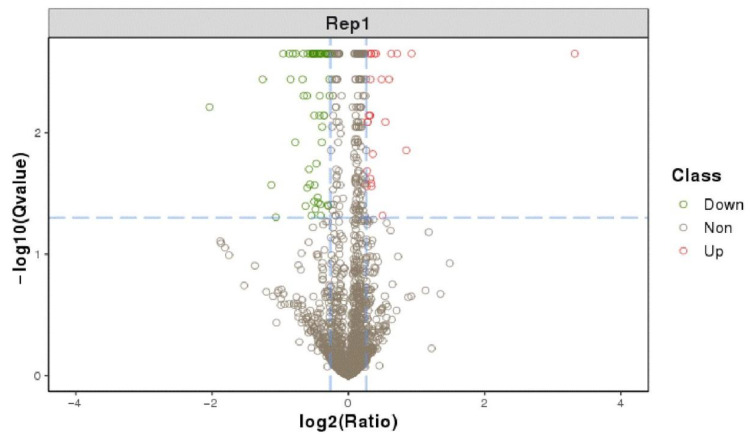
Differentially expressed protein volcano graph. The X-axis is the protein difference multiple (take log2) and the Y-axis is the corresponding −log10 (Q-value).

In total, 91 significant differentially expressed proteins were identified using fold change > 1.2 and Q-value < 0.05 criteria, of which 26 proteins were significantly up-regulated and 65 significantly down-regulated (Figure 5). The most up-regulated protein was YhaK, which belonged to the Pirin family. The AE81Δ*ygeK/*AE81 ratio was 10. The most down-regulated protein was AdiC which was an arginine: agmatine antiporter (Table S1).

### 3.6. KEGG Pathway Annotation Analysis of Differentially Expressed Proteins

**Figure 6:**
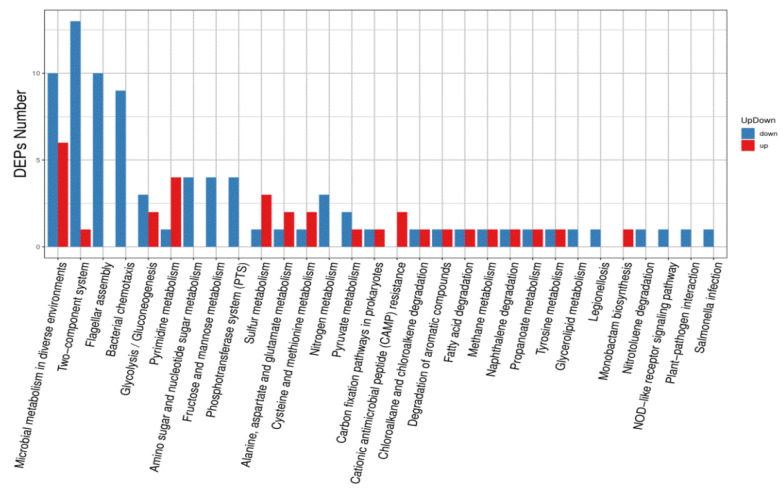
Up-regulated and down-regulated differentially expressed protein pathway classification.

**Figure 7:**
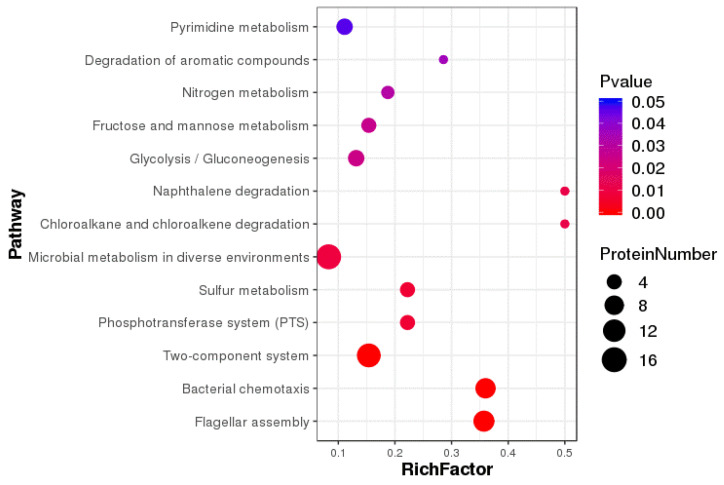
Enrichment pathways show significant differentially expressed proteins.

KEGG pathway annotation analyses showed that differentially expressed proteins were mainly enriched in microbial metabolism in diverse environments, two-component systems, flagellar assembly, bacterial chemotaxis, glycolysis/gluconeogenesis, and pyrimidine metabolism, amino sugar and nucleotide sugar metabolism, and fructose and mannose metabolism (Figure 6). Differentially expressed proteins were most prevalent in microbial metabolism in diverse environments, two-component systems, bacterial chemotaxis, and flagellar assembly (Figure 7). 

## 4. Discussion

APEC is reportedly a potential zoonotic pathogen that transfers virulence and resistance genes to human ExPEC; therefore, developing effective vaccines or new drug targets is imperative [25]. In our previous study, *ygeK* significantly affected APEC flagella formation, bacterial motility, serum sensitivity, adhesion, and virulence via different functional pathways [16]. Here, we report that inactivation of the transcriptional activator *ygeK* decreased biofilm formation and reduced bacterial resistance to acid and oxidative stresses. 

Biofilm formation is a protected growth mode that enables pathogen survival in hostile environments [26,27]. Several transcriptional regulators are involved in APEC biofilm formation, including *basSR*, *phoP*, *mcbR*, and *cpxA* [11,12,13,28]. In bacteria, common regulators, extracellular polymeric production, and biofilm heterogeneity are all central responses and contributors to oxidative stress [29]. For example, *Salmonella* biofilms cultured in vitro are also tolerant to H_2_O_2_ [30]. In APEC, inactivation of *ibeA* decreased biofilm formation and *ibeA* can confer increased H_2_O_2_ resistance to APEC [31,32]. In our study, *ygeK* inactivation decreased biofilm formation and reduced oxidative responses in APEC. Inactivation of *mcbR* increased APEC’s biofilm formation but decreased APEC’s resistance to H_2_O_2_ stress [13]. Deletion of *waaL* increased biofilm formation and reduced resistance to oxidative and alkali environmental stress [33]. Therefore, we hypothesize that these genes affecting biofilm formation and H_2_O_2_ resistance are involved in different pathways. H_2_O_2_ readily crosses bacterial membranes and enters the cytoplasm where it forms hydroxyl radicals that damage DNA, proteins, and lipid membranes [31]. In APEC, oxidative responses exert important roles during pathogenic processes in hosts. We provide a greater understanding of how APEC is recalcitrant to oxidative stress during chronic infection.

Acid stress is also a typical environmental stress [34]. Microorganisms have developed sophisticated physiological and molecular mechanisms to survive under acid stresses such as the sophisticated acid-resistant systems of *E. coli*. One acid-resistant system, composed of Glutaminease YbaS and the Glu-GABA antiporter GadC, helps *E. coli* survive in extremely acid environments [35,36,37]. There are also other factors influencing acid stress. For example, deletion of *yfcO* decreased survival under acidic stress conditions [38]. The mutation of *ybjX* decreased resistance to environmental stress (alkaline and acid) [23]. Without *ygeK*, the acid resistance of APEC decreased, with our proteomics data showing that GadC protein expression was down-regulated, indicating *ygeK* may affect APEC mediated acid resistance via GadC. This result highlights how *ygeK* has a role in developing resistance to acid stress.

In this study, we reported that deletion of *ygeK* decreased biofilm formation ability and reduced resistance to environmental stress. When combined with our previous findings, we hypothesize that *ygeK* plays a vital function in APEC processes. In addition, we analyzed *ygeK* function in APEC using proteomics, which not only enriches the APEC network but also unravels APEC pathogenic mechanisms for the generation of alternative control strategies for APEC virulence. In the future, substrate interaction with *ygeK* should be investigated to further investigate the mechanism.

## 5. Conclusions

This study proved that transcriptional regulator *ygeK* influences APEC biofilm formation, resistance to H_2_O_2_, and acid resistance. In addition, we provide a greater understanding of how APEC is recalcitrant to oxidative stress during chronic infection, highlight how *ygeK* has a role in developing resistance to acid stress, and elucidate its regulatory network. These results define the critical role of *ygeK* in APEC, guiding the search for new drug targets and vaccines.

## Data Availability

iTRAQ-based proteomic sequencing data were deposited into the iProX integrated proteome resources with Project ID IPX0004369000.

## Supplementary Materials

The following supporting information can be downloaded at: https://www.mdpi.com/article/10.3390/ani12091160/s1, Table S1: The differentially expressed proteins between AE81 and AE81Δ*ygeK*.
